# Habitat, seasonal temperature and collection year drive variable germination responses in the endangered plant *Harperocallis flava*

**DOI:** 10.1093/conphys/coaf079

**Published:** 2025-11-22

**Authors:** Amber G Gardner, Héctor E Pérez

**Affiliations:** Department of Environmental Horticulture, University of Florida, 2043 IFAS Research Drive, Gainesville, FL 32611, USA; Department of Environmental Horticulture, University of Florida, 2043 IFAS Research Drive, Gainesville, FL 32611, USA

**Keywords:** Conservation, intra- and interpopulation variation, plant nutrition, plasticity, seed functional traits, selection pressure, soil nutrients, spatiotemporal variation

## Abstract

Plant conservation programs strive to integrate information from various life-history stages of focal species when developing holistic recovery strategies. Therefore, in-depth knowledge of the seed-to-seedling transition, a crucial phase that begins with the germination process, provides key perspectives that support recovery. Analyses of seed functions (e.g. germination timing) and related traits (e.g. germination rate, temperature requirements, stress tolerance) in response to selective pressures (e.g. temperature) can fill key knowledge gaps for the seeds of most at-risk plants. Here, we investigated the germination ecology of *Harperocallis flava*, a rare, globally imperilled, federally and state endangered (government protected) species from Florida, USA. We tested the germination of fresh seeds from three habitats collected over 3 years following exposure to simulated seasonal temperatures of winter (day/night temperatures of 20/10°C), early spring/late fall (25/15°C), early fall/late spring (30/20°C), or summer (35/25°C). We quantified the germination response of *H. flava* seeds to habitat of origin, seasonal temperature and collection year to determine how these factors influence germination dynamics and to inform seed-based conservation of this and other rare species. Considerable variation in germination responses was observed among the temperature treatments, seed collection years and habitats. Germination tended to be lower at simulated summer temperatures and higher at winter and late fall/early spring temperatures, which suggests that germination in the wild likely occurs in the period following natural shedding while temperatures are below 30°C (i.e. late fall through early spring). Moreover, the spatiotemporal variation of the germination responses highlights the value of basing conservation recommendations on multi-year, multi-population seed biology research when possible.

## Introduction

Plant conservation programs worldwide are actively engaged in collecting seed biology information of focal taxa to support and expand seed-based conservation strategies. However, acquiring baseline data that can inform management decision-making for threatened species can be challenging. Factors that exacerbate the knowledge gap may include a lack of personnel with seed biology training, coupled with difficult or unknown species biology; an emphasis on in situ conservation rather than an integrated in- and ex situ approach; limited political or financial support; challenges accessing plant populations; limitations on seed availability; and the sheer number of species requiring standard seed biology research (Hay and Probert, 2013; Havens *et al.*, 2014; Ribeiro *et al.*, 2016; Heywood, 2017; Breman *et al.*, 2021; Mattana *et al.*, 2022). Research focusing on plant regeneration from seeds and environmental cues that influence this process, can provide conservation practitioners with important insights into the reproductive barriers faced by threatened species. In particular, the seed-to-seedling transition is widely regarded as the most stress-vulnerable life history stage for spermatophytes (Parker *et al.*, 2008). Knowledge about seed responses to abiotic signals during the seed-to-seedling transition can be utilized to augment in- and ex situ conservation activities like ecosystem restoration, production of plant materials for out-planting and germplasm banking (Falk *et al.*, 1996; Guerrant *et al.*, 2004).

Seeds continually use many environmental cues to ensure that germination occurs under conditions that favour seedling establishment (Baskin and Baskin, 2014; Huang *et al.*, 2015). Temperature sensing is a primary factor influencing dormancy alleviation and germination in hydrated seeds. Temperature directly regulates the rates of biochemical reactions, metabolism and physiological processes that lead to dormancy break and eventual germination (Hills and van Staden, 2003; Côme and Corbineau, 2006; Baskin and Baskin, 2014). Temperature also controls the rate and uniformity of germination in non-dormant seeds (Probert, 2000; Hills and van Staden, 2003; Baskin and Baskin, 2014; Huang *et al.*, 2015). Thus, gradual changes in temperature can provide seeds with seasonal cues for dormancy break or germination (Huang *et al.*, 2015). Additionally, daily temperature fluctuations typically indicate the presence of a vegetation gap or depth of seeds in the soil profile (Fenner and Thompson, 2005). For example, extreme temperature fluctuations are often experienced near the soil surface, while more constant temperatures reflect deeper burial. Likewise, very high temperatures experienced during fires can stimulate germination in some species through heat shock (Fenner and Thompson, 2005).

The habitat in which seeds develop also plays a major role in subsequent dormancy alleviation and germination responses (Penfield and MacGregor, 2017; references therein). Signals such as altitude, light quality and quantity, nutrient availability, soil chemical and physical properties and temperature, are perceived by maternal tissues prior to and during gametic and zygotic development (Roach and Wulff, 1987; Baskin and Baskin, 2014; Penfield and MacGregor, 2017; references therein). This information can be passed down from mother to progeny during seed development, manifesting as variations in seed traits (e.g. hormone balance, morphology) that influence the germination process (Meyer and Monsen, 1991; Mira *et al.*, 2017; Tudela-Isanta *et al*., 2018; Fernández-Pascual *et al.*, 2019). These maternal, and sometimes paternal, effects can persist across multiple generations (Baskin and Baskin, 2014; Penfield and MacGregor, 2017; references therein).

In addition to the cues passed from parent plants to offspring and those acquired during seed development, plants and seeds possess some flexibility in their response to environmental variations through the well-documented phenomenon of phenotypic plasticity (Fenner and Thompson, 2005; Wainwright and Cleland, 2013; Xue and Leibler, 2018; Fox *et al.*, 2019). As sessile organisms, plants must express plasticity in their responses to external stimuli for survival (Galloway and Etterson, 2007; Fox *et al.*, 2019). This plasticity allows plants to respond relatively quickly to constantly fluctuating environmental conditions (Fernández-Pascual and Jiménez-Alfaro, 2014; Fox *et al.*, 2019). For instance, germination plasticity can result in faster or slower germination across a seed population that translates into temporal distribution of seedling establishment in response to variable inter-annual environmental conditions (Wainwright and Cleland, 2013; Baskin and Baskin, 2014).

Understanding the influence of temperature and habitat on germination, and how these effects may vary over time, is of paramount importance to plant conservation efforts. Seed collection and propagation plans may be improved with an understanding of the degree of inter-site germination plasticity in a species occurring across different habitats (Bischoff *et al.*, 2006). A multi-year approach to studying germination plasticity is essential, as single-year studies on seeds of wild species may result in biased estimates of germination performance that could misinform conservation practice. Time-separated seed collections can capture important fluctuations in germination responses due to phenotypic plasticity or local adaptation while providing a more comprehensive picture of focal species germination ecology (Garwood, 1983; Everingham *et al.*, 2021; Chidumayo, 2024).

Baseline reproductive ecology information takes on increased significance as conservation practitioners plan for climate change mitigating approaches, including inter situ conservation, assisted migration and adaptive management (Burney and Burney, 2007; Runge, 2011; Zimmer *et al.*, 2019). However, given limitations described previously, crucial seed biology information may not be promptly available, as in the case of the globally imperilled plant *Harperocallis flava* [Tofieldiaceae]. There was a 43-year gap between listing *H. flava* for governmental protections (USFWS, 1979; NatureServe, 2023) and inclusion of robust seed biology information in the species recovery plans (Gardner, 2017, 2021; USFWS, 2022). Dedicated seed biology research programs with a conservation emphasis can therefore short-circuit the time it takes to deliver much needed knowledge.

In this paper, we focus on the relative impacts of habitat of origin, environmental cues and multi-year seed sampling on germination dynamics with an emphasis on *H. flava* seed biology. Reports suggest that *H. flava* genetic diversity is extremely low and populations are declining (Walker and Silletti, 2005; USFWS, 2016). Decreased precipitation and suppression of regenerative, landscape- or habitat-scale fires are listed as potential causes for the observed declines (Walker and Silletti, 2005; USFWS, 2016). Seedling recruitment (i.e. survival and incorporation of new individuals into a population) appears to be highly limited and has not been observed (Walker and Silletti, 2005; USFWS, 2016). Seeds dispersed into the environment are subject to many fates including germination, predation, incorporation into the soil seed bank, or death (Long *et al.*, 2015). In a previous study of *H. flava* seedling emergence in the natural habitat, we found very low seedling emergence (0–17%) and even lower survival (0–2%) possibly resulting from inadequate soil moisture during the study period (Gardner, 2017). We have also observed pre-dispersal predation of immature seeds and capsules by orthopterans (Gardner, 2021) which may further reduce the opportunities for seedling recruitment (Fenner and Thompson, 2005). Less than 50% of the existing *H. flava* populations are expected to persist into the next decade without intervention (USFWS, 2016). Thus, *H. flava* conservation planning documents (USFWS, 1983, 2009, 2016, 2022) call for investigations into the seed biology and germination ecology of this species to better understand a vital yet potentially limiting stage in the plant life cycle*.*

This study builds on our previous research concluding that *H. flava* seeds have low seedling emergence and survival in the field (Gardner, 2017), can form short-term persistent soil seed banks (Fenner and Thompson, 2005; Gardner, 2021), and can possess morphological or morphophysiological dormancy (Baskin and Baskin, 2014; Gardner, 2021). Germination is inhibited by darkness and high, simulated summer temperatures but promoted by fall through spring temperatures, with optimal germination at alternating 25/15°C or constant 25°C (Gardner, 2017, 2021). Therefore, our primary questions were: (i) to what extent do habitat of origin, seasonal temperatures and collection year modify *H. flava* germination responses; and (ii) how can key findings inform seed-based conservation? Our objectives were to contribute a broader understanding of factors that influence germination responses; recommend a template for assessing variable germination responses of other rare species; and provide information that could enhance achievement of *H. flava* recovery goals (USFWS, 1983, 2009, 2016, 2022).

## Materials and Methods

### Species and habitat information

*Harperocallis flava* is a rare, endemic species found only in a small area of northwest Florida, USA. Anthesis typically occurs from April through early June, and capsules develop throughout June and into October. Seed dispersal via capsule dehiscence occurs primarily in late September through October. *Harperocallis flava* seeds were collected at maturity from three different habitats (ca. 3 to 8 km between habitats) a seepage bog, a forest ecotone and a roadside right-of-way. We refer to germplasm from these habitats as bog, forest and roadside seeds.

The bog environment is characterized by forbs, graminoids and various carnivorous plants (e.g. *Sarracenia* spp., *Drosera* spp. and *Pinguicula* spp.) with virtually no tree canopy cover. The soils are moderately acidic (pH 5.6–5.8), nutrient poor (Table S1; Gardner, 2017) and remain saturated with standing water for most of the year. In contrast, the forest environment occurs in an ecotone between a cypress (*Taxodium ascendens*) swamp and pine (*Pinus* spp.) flatwoods. The vegetation structure includes a dense groundcover layer of various herbaceous and graminoid species, with an open-canopy pine overstory, and a well-developed shrubby midstory, primarily consisting of swamp titi (*Cyrilla racemiflora*). This area experiences seasonal inundation followed by pronounced dry periods. Soils are very acidic (pH 3.9–4.0), low in nutrients and contain 5–6% organic matter content (Table S1; Gardner, 2017). The roadside environment occurs along a right-of-way that is mowed annually, which has contributed to a dominant groundcover layer of herbaceous and graminoid species, including the non-native, planted bahiagrass (*Paspalum notatum*). *Harperocallis flava* plants typically occur on the upper slope of the roadside swale, and the soil in the vicinity of the plants tends to be dry to moist (Gardner, 2017, 2021). Soil pH was moderately acidic (5.2–5.6) with moderate to high levels of magnesium (35–81 mg/Kg) and moderate levels of potassium (Table S1; Gardner, 2017).

Lightning strikes, vandalism and wildlife destroyed our environmental sensors located within the habitats. Nonetheless, we conducted a retrospective analysis of temperature and precipitation with observations from a spatial dataset (PRISM Climate Group, 2014) to gain some perspective on possible differences in climatic conditions between the habitats. We input site coordinates to the PRISM Data Explorer tool then requested monthly precipitation and minimum, mean and maximum temperatures for each habitat based on 4 km grid resolution.

### Ethical declarations

Seed capsules were collected under the following permits: TE47720B-0 (United States Fish & Wildlife Service), FS-2400-008 (United States Department of Agriculture Forest Service) and 1331 (Florida Department of Agriculture and Consumer Services Division of Plant Industry).

### Collection of capsules from wild plants and seed handling: 2018–2020

To reduce herbivory, we located and covered developing *H. flava* fruits with 60 × 70 mm nylon drawstring bags (mesh size ≈ 0.2 mm) within each habitat during June 2018, 2019 and 2020. Mature brown capsules were hand-collected from plants at the time of natural dispersal in late September (2019) or early October (2018 and 2020; Table S2). The diminutive (i.e. 2.37 ± 0.30 mm long × 0.26 ± 0.03 mm wide; mean ± SD) seeds were removed from capsules in the lab. Any non-filled, damaged, or visibly infected seeds and capsules were excluded from experiments. Seeds were stored under laboratory conditions (≈ 22–25°C; 40–50% relative humidity) in Petri dishes for 3 to 13 days (Table S2).

### Germination of collected seeds at simulated seasonal temperatures

Germination was tested within 2 weeks of collection (Table S2) by exposing seeds to various alternating temperatures within germination chambers (I-30VL; Percival Scientific, Perry, IA, USA). The alternating temperature regimes were modified from Pérez and Kettner (2013) and are based on average monthly minimum and maximum temperatures over a 30-year period across Florida. These temperatures were chosen to simulate seasonal temperatures for winter (20/10°C; December–March), late fall or early spring (25/15°C; ca. November–December, or March–April respectively), early fall or late spring (30/20°C; ca. September–October, or May–June respectively) and summer (35/25°C; June–September). Temperatures were checked with thermometers periodically to ensure they matched the set temperatures. We examined germination under a range of simulated seasonal conditions to gain a better understanding of *H. flava* germination ecology, determine thermal limits for germination and provide insight into how germination patterns might change over time. Temperatures and photoperiod alternated every 12 hours within all chambers. Cool white fluorescent bulbs (Philips FT17T8/TL841 Alto) provided a photosynthetic photon flux density of 57 ± 7 μmol m^−2^ s^−1^ (mean ± SD) at seed level. Illumination coincided with the higher temperatures described above.

We randomly selected samples of ~400 intact seeds collected from each habitat in each collection year (Table S2). Seed lots were then divided into sub-samples of 25 to 38 seeds and sown on blotter paper (Anchor Paper, St. Paul, MN, USA) in clear polystyrene germination boxes (≈11 × 11 × 3.5 cm; Hoffman Manufacturing, Corvallis, OR, USA). Blotter paper was moistened with enough sterilized (autoclave: 40 min, 121°C, 117 kPa), distilled, de-ionized water to saturate the blotter, without leaving a film of water over the seeds. Supplemental water was added as needed throughout the experiment.

We randomly assigned four boxes from each habitat and collection year to each of the simulated seasonal temperatures. We conducted germination checks daily for 35 days, which was determined by previous experiments to be sufficient time for germination at most temperatures (Gardner, 2017). Seeds consumed by bacterial or fungal pathogens were removed from the experiment and counted as censored observations. *Harperocallis flava* seeds do not follow the common pattern of radicle emergence as the first sign of visible germination. Instead, the radicular end of the embryo emerges from the testa prior to development of any roots, while the cotyledonary portion remains inside the seed. Occasionally, the testa will split laterally and the cotyledonary portion of the embryo emerges first. Therefore, germination was defined as any embryonic tissue emerging from the testa. Similar non-standard germination patterns have been documented for numerous species (Baskin and Baskin, 2014, 2021).

**Figure 1:**
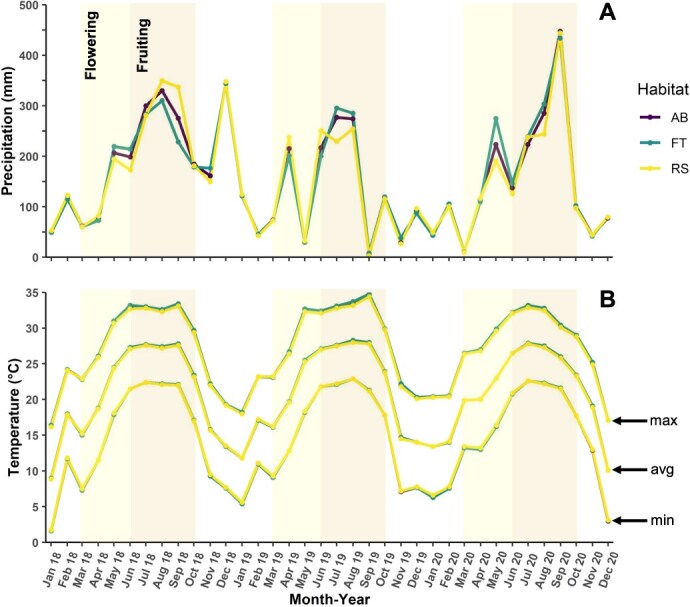
Total monthly precipitation and average temperature data during 2018, 2019, and 2020 for three seed collection sites: bog (AB), forest (FT), and roadside (RS) (PRISM Climate Group).

**Figure 2:**
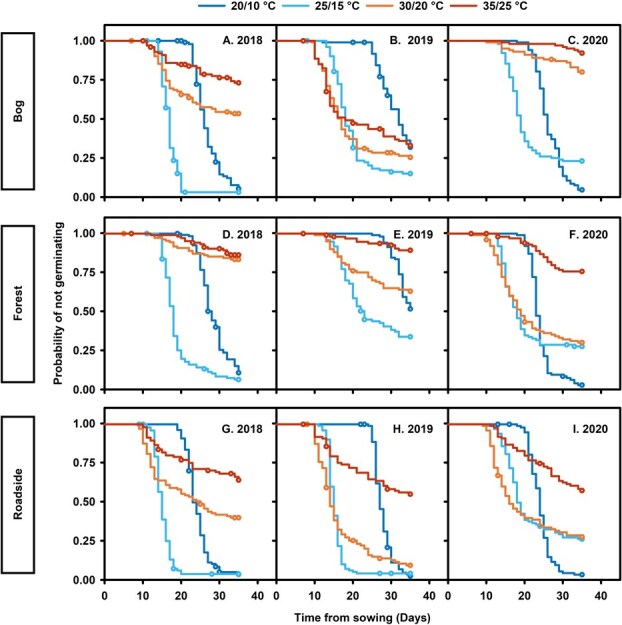
Patterns of *Harperocallis flava* seed germination stratified by seed habitat (bog, forest, and roadside), seed collection year (2018, 2019, and 2020), and seasonal temperature. Open circles represent censored observations. Ninety-five percent confidence intervals are omitted for clarity.

Because Tetrazolium testing was unreliable for *H. flava* (A. Gardner unpublished work), post germination seed viability was instead assessed using a cut test following the approach of Baskin and Baskin (2014) and Ooi *et al.* (2004). For the 2018 viability analyses, all non-infected and non-germinated seeds were dissected under a stereomicroscope. Embryos displaying obvious growth and greening were classified as viable, while white, underdeveloped embryos were classified as non-viable. Although Baskin and Baskin (2014) consider firm white embryos viable, we used a more conservative criterion because embryo greening initially provided a more definitive indication of *H. flava* seed viability. However, a year-long germination study (Gardner, 2021) suggested that some white embryos were likely dormant or thermo-inhibited. Accordingly, for the 2019 and 2020 germination experiments, all remaining non-germinated seeds were moved to optimal temperatures (25/15°C) for 2 to 3 additional weeks to determine viability. Seeds that germinated or contained green embryos following this period were counted as viable, whereas small non-green embryos were considered non-viable (Gardner, 2021).

### Statistical analysis

We used time-to-event analysis to assess germination in response to the temperature, habitat and collection year variables. Germination was coded as 1 and censored events (i.e. contaminated, lost, or non-germinated seeds at the end of the experiment) were coded as 0. We estimated survivor functions, which were stratified by the covariates of interest, using Kaplan–Meier methods. Then, we used the estimates to generate median germination time (*t*_50_) and germination rate (i.e. 1 ∙ *t*_50_^−1^). In cases where the probability did not reach 0.50, the median germination time and germination rates could not be estimated. Finally, we used the log-rank statistic to test the hypothesis of no difference in germination patterns when stratifying the dataset by habitat, temperature and year.

We modelled the likelihood of germination with Cox regression and utilized the exact method to account for tied event times (Allison, 2010). We assessed for violations of the proportional hazards assumption graphically and with Schoenfeld residuals (Allison, 2010). We incorporated time-dependent covariates (e.g. temperature × day) into the models when violations of the PH assumption occurred. We created orthogonal linear contrasts to examine changes in the likelihood of germination associated with interactions between our variables of interest (Allison, 2010; Pérez and Kane, 2017). We assessed climate and seed viability data with analysis of variance (ANOVA) and Kruskal–Wallis test on Wilcoxon mean scores, respectively, and utilized the Dwass–Steel–Critchlow–Fligner method for post-hoc analysis of viability data. All statistical analyses and data visualizations were conducted in SAS (v 9.4, SAS Institute, Cary NC, USA) and SigmaPlot (v 14.5, Systat Software, Inc., Palo Alto, CA, USA), respectively.

## Results

### Climate

Retrospective analysis of climate revealed that there were some differences in precipitation between the three sites during the flowering and seed development periods across years (Fig. 1A). For example, in 2019, precipitation was lower across all habitats from August through September, during the latter stages of fruit development, compared to 2018 and 2020 (Fig. 1A). However, ANOVA did not detect a statistically significant habitat × year interaction during the flowering (*F*_4,18_ = 0.11, *P* = 0.98) or seed development (*F*_4,36_ = 0.04, *P* = 0.99) phenophases. Likewise, the main effects of habitat and year were not statistically significant during the flowering period (habitat *F*_2,22_ = 0.02, *P* = 0.98; year *F*_2,22_ = 0.00, *P* = 0.99) or seed development (habitat *F*_2,40_ = 0.00, *P* = 0.99; year *F*_2,40_ = 2.32, *P* = 0.11).

Monthly minimum, average and maximum temperatures during flowering and seed development were nearly identical (Fig. 1B). Analysis of variance detected no statistical differences in minimum (flowering *F*_8,8_ = 0.66, *P* = 0.71; seed development *F*_8,8_ = 0.92, *P* = 0.54), average (flowering *F*_8,8_ = 0.59, *P* = 0.77; seed development *F*_8,8_ = 0.44, *P* = 0.86) and maximum (flowering *F*_8,8_ = 0.55, *P* = 0.79; seed development *F*_8,8_ = 0.26, *P* = 0.96) temperatures during the study period.

### Germination dynamics

Stratified Kaplan–Meier plots revealed statistically significant differences in germination patterns within and across the habitats, simulated seasonal temperatures and collection years (log-rank ${\chi}_{35}^2$ = 1791.52, *P* < 0.0001; Fig. 2). For example, seeds collected in 2018 from all three habitats displayed rapid and nearly complete germination (84–93%) at 20/10 and 25/15°C, with 25/15°C being the optimal germination temperature (i.e. highest germination percentage in the shortest time; Fig. 2A, D, G; Table 1). The germination patterns for all 2018 seeds exposed to 30/20 and 35/25°C indicated germination sensitivity characterized by higher probabilities of not germinating and slower, less uniform and reduced total germination (Fig. 2A, D, G; Table 1). However, seeds collected from the forest habitat exhibited greater sensitivity to 30/20 and 35/25°C than the bog or roadside seeds (Fig. 2A, D, G).

**Table 1:** Germination (germ.) parameters for *Harperocallis flava* seeds collected during 2018, 2019 and 2020 from three habitats

Temp. (°C)	Seed lot/collection year	*n*	Final germ. (%)	Lag (d) (95% CI)	Median germ. time (*t_50_*) (d) (95% CI)	Germ. rate (1/*t*_50_)	Post germ. viability (%)^a^
20/10	AB18	104	84	22 (21, 23)	26 (25, 27)	0.04	87
	FT18	122	87	22 (21, 24)	28 (27, 30)	0.04	96
	RS18	112	93	20 (19, 20)	24 (23, 25)	0.04	95
25/15	AB18	105	87	14 (14, 15)	17 (16,17)	0.06	88
	FT18	112	91	15 (14, 15)	18 (17,18)	0.06	94
	RS18	104	93	13 (12, 13)	15 (14,15)	0.07	95
30/20	AB18	104	45	12 (11, 13)	—	—	52
	FT18	110	16	16 (12, 20)	—	—	20
	RS18	106	59	10 (9, 10)	25 (19, —)	0.04	62
35/25	AB18	103	25	12 (11, 13)	—	—	29
	FT18	106	13	20 (13, 26)	—	—	18
	RS18	106	35	11 (10, 11)	—	—	41
20/10	AB19	102	62	22 (15, 28)	32 (30,34)	0.03	84
	FT19	104	48	27 (26, 29)	—	—	90
	RS19	101	93	25 (24, 26)	27 (27,28)	0.04	96
25/15	AB19	103	83	14 (13, 14)	18 (17,20)	0.06	87
	FT19	98	64	15 (13, 16)	23 (20,30)	0.04	73
	RS19	103	95	13 (11, 14)	15 (15,16)	0.07	95
30/20	AB19	115	74	10 (9, 10)	17 (15,18)	0.06	83
	FT19	101	37	13 (11, 14)	—	—	55
	RS19	105	87	10 (10, 10)	14 (14,15)	0.07	90
35/25	AB19	115	64	10 (10, 10)	18 (14,28)	0.06	85
	FT19	102	10	17 (13, 22)	—	—	71
	RS19	100	43	11 (10, 11)	—	—	84
20/10	AB20	102	95	20 (19, 22)	26 (25,27)	0.04	98
	FT20	101	96	20 (19, 20)	23 (23,24)	0.04	97
	RS20	100	94	20 (18, 22)	24 (24,25)	0.04	96
25/15	AB20	103	77	13 (12, 14)	19 (18,20)	0.05	90
	FT20	100	72	13 (13, 14)	18 (17,19)	0.06	92
	RS20	100	73	12 (11, 14)	19 (18,20)	0.05	87
30/20	AB20	101	20	17 (11, 24)	—	—	96
	FT20	102	69	9 (7, 11)	19 (17,23)	0.05	94
	RS20	101	71	10 (9, 11)	16 (15,24)	0.06	98
35/25	AB20	102	8	26 (18, 34)	—	—	99
	FT20	103	23	17 (12, 22)	—	—	94
	RS20	100	42	13 (12, 13)	—	—	94

Seeds were exposed to simulated seasonal temperatures (temp.) for 35 days. The abbreviations AB, FT, and RS represent the bog, forest and roadside habitats. Lag time describes the mean number of days from imbibition to germination. Median germination time (*t*_50_) refers to the 50th percentile of germination or the smallest event time where the probability of germinating earlier is greater than 0.50. Germination rate is the inverse of the median germination time (1 · *t*_50_^−1^). Total post germination seed viability consisted of germinated and non-germinated but viable seeds.

a Viability was determined using a conservative approach in 2018 which underestimated post germination viability especially at higher temperatures.

Patterns shifted for bog and forest seeds collected in 2019. Seeds exposed to the lowest temperatures displayed reduced germination compared to 2018 seeds or 2019 roadside seeds (Fig. 2B, E, H). Forest seeds displayed much greater reductions in germination capacity at lower temperatures than the bog seeds (Fig. 2B, E). Another key difference in 2019 was that exposure to 30/20°C resulted in more rapid and complete germination for bog and roadside seeds compared to 2018 and 2020 seeds at the same temperature. Interestingly, bog seeds exposed to 35/25°C in 2019 displayed the highest germination (64%) at this temperature compared to seeds exposed to 35/25°C from all other habitats and collection years (Fig. 2, Table 1). While an optimum germination temperature was less evident for bog seeds in 2019, seeds exposed to 25/15°C exhibited the highest proportions of germination compared to other temperatures, though this difference was less pronounced in 2019 than in 2018 (Fig. 2B, E, H; Table 1). Roadside seeds collected in 2018 and 2019 displayed more robust germination patterns compared to the bog and forest seeds collected in the same years (Fig. 2A, B, D, E, G, H).

**Table 2:** Summary of joint tests for the extended Cox regression models for *Harperocallis flava* seeds during 2018, 2019 and 2020 from three habitats (bog, forest and roadside) and exposed to simulated seasonal temperatures representing winter (20/10°C), late fall/early spring (25/15°C), early fall/late spring (30/20°C) and summer (35/25°C) conditions across Florida for 35 days

Effect	Degrees of freedom	Wald χ2	*P*
Habitat	2	16.47	0.0003
Collection year	2	1.561	0.4580
Temperature	3	508.64	<0.0001
Habitat × collection year	4	53.02	<0.0001
Habitat × temperature	6	14.37	0.0258
Collection year × temperature	6	146.71	<0.0001
Habitat × collection year × temperature	12	115.19	<0.0001
Habitat × day	1	4.58	0.0323
Temperature × day	1	723.29	<0.0001

We incorporated time-dependent covariates (e.g. temperature × day) into the models when violations of the proportional hazards assumption occurred.

In 2020, germination under the previously optimal 25/15°C regime was similarly reduced for seeds from all habitats, with final germination reaching 72% to 77%. Alternatively, final germination (94–96%) at 20/10°C was the highest across habitats (Fig. 2C, F, I; Table 1). Germination patterns differed for forest seeds in 2020, as germination capacity improved considerably across most temperatures compared to previous years (Fig. 2D, E, F; Table 1). The germination responses of the roadside seeds were generally more consistent across collection years and were more resilient to high-temperature stress than seeds from most of the other habitats and collection years (Fig. 2; Table 1). Germination rates indicated that the optimum germination temperature for seeds collected from the roadside fluctuated between 25/15°C and 30/20°C depending on the year of collection (Fig. 2G-I; Table 1).

### Germination modelling and interaction analyses

**Figure 3:**
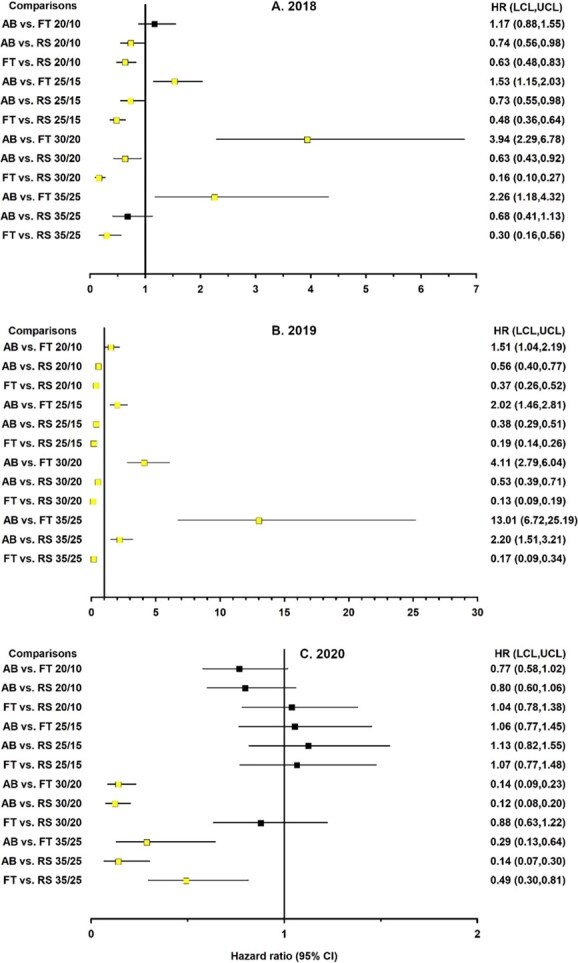
Forest plot depicting linear orthogonal contrasts of the likelihood of germination for *Harperocallis flava* seeds between three habitats (bog (AB), forest (FT), and roadside (RS)) across seasonal temperatures for different collection years. Squares and horizontal lines represent the hazard ratios and 95% confidence limits, respectively. The vertical line represents the reference line equal to 1.00 for determination of statistical significance. Confidence limits overlapping 1.00 or the reference line denote a non-statistically significant difference. Light and dark squares also denote significant and non-significant hazard ratios, respectively. Squares to the right of the reference line indicate that the first factor in a comparison was associated with a greater likelihood of germination than the second factor. Squares to the left of the reference line indicate that the second factor in the comparison was associated with a higher likelihood of germination, in which case taking the reciprocal of the hazard ratio reverses the comparison (e.g. AB vs. RS becomes RS vs. AB).

**Figure 4:**
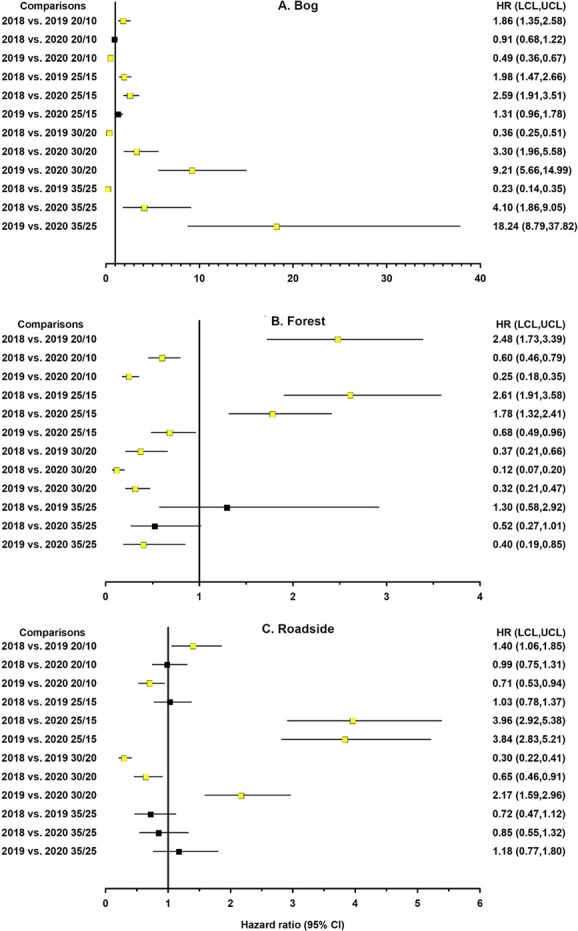
Forest plot depicting linear orthogonal contrasts of the likelihood of germination for *Harperocallis flava* seeds between collection years across seasonal temperatures for different habitats: (A) bog, (B) forest ecotone, and (C) roadside. Squares and horizontal lines represent the hazard ratios and 95% confidence limits, respectively. The vertical line represents the reference line equal to 1.00 for determination of statistical significance. Confidence limits overlapping 1.00 or the reference line denote a non-statistically significant difference. Light and dark squares also denote significant and non-significant hazard ratios, respectively. Squares to the right of the reference line indicate that the first factor in a comparison was associated with a greater likelihood of germination than the second factor. Squares to the left of the reference line indicate that the second factor in the comparison was associated with a higher likelihood of germination, in which case taking the reciprocal of the hazard ratio reverses the comparison (e.g. 2018 vs. 2019 becomes 2019 vs. 2018).

**Figure 5:**
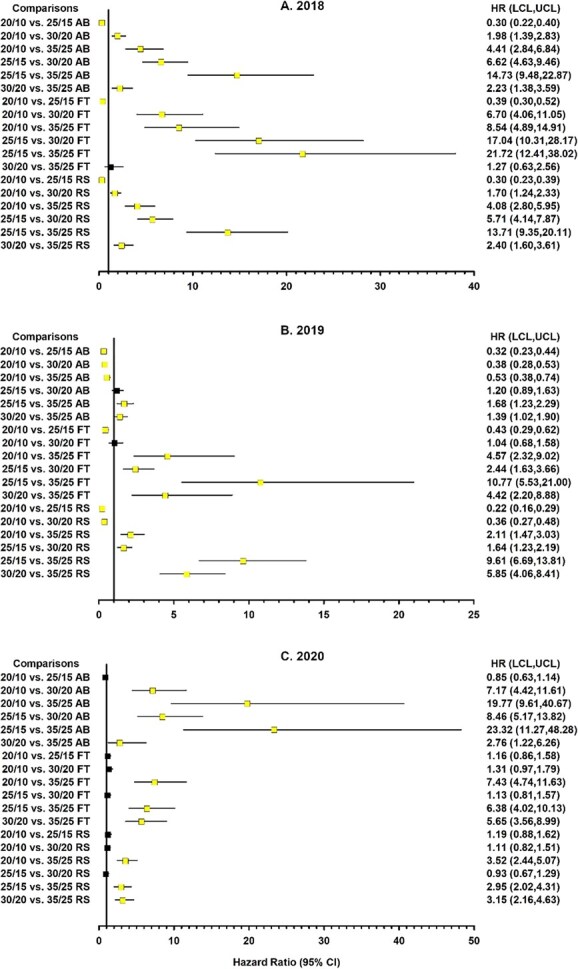
Forest plot depicting linear orthogonal contrasts of the likelihood of germination for *Harperocallis flava* seeds between seasonal temperatures across three habitats (bog (AB), forest (FT), and roadside (RS)) and different collection years. Squares and horizontal lines represent the hazard ratios and 95% confidence limits, respectively. The vertical line represents reference line equal to 1 for determination of statistical significance. Confidence limits overlapping 1.00 or the reference line denote a non-statistically significant difference. Light and dark squares also denote significant and non-significant hazard ratios, respectively. Squares to the right of the reference line denote that the first factor in a comparison was associated with a greater likelihood of germination than the second factor. Squares to the left of the reference line denote that the second factor in the comparison was associated with a higher likelihood of germination, in which case taking the reciprocal of the hazard ratio reverses the contrast comparison (e.g. 20/10 vs. 25/15°C becomes 25/15 vs. 20/10°C).

Cox regression detected a statistically significant three-way interaction between habitat, seed collection year and seasonal temperature (Wald ${\chi}_{12}^2$ = 115.1914; *P* < 0.0001; Table 2). Therefore, we separated linear orthogonal contrasts into three groups that compared the effects of: (i) habitats across seasonal temperatures over different collection years (Fig. 3); (ii) collection years across seasonal temperatures over different habitats (Fig. 4); and (iii) seasonal temperatures across habitats over different collection years (Fig. 5).

Orthogonal contrasts revealed that the influence of habitat on the likelihood of germination varied considerably across the simulated seasonal temperatures and collection years. Moreover, the magnitude of the habitat effect (i.e. hazard ratios [HR]) tended to increase as temperatures increased (Fig. 3). Note: the reported hazard ratios represent comparisons in one direction; taking the reciprocal (1/HR) reverses the comparison (e.g. AB vs RS becomes RS vs AB; Figs. 3-5) and provides the corresponding hazard ratio for that comparison. Roadside seeds tended to exhibit higher and faster germination than seeds from other habitats, especially in the first two collection years. The likelihood of germination for seeds collected in 2018 and 2019 was about 1.4 to 7.7 times higher for roadside seeds than for bog or forest seeds and about 1.5 to 13.0 times greater for bog seeds than for forest seeds (Fig. 3A, B). These patterns suggest physiological variation among seeds from different habitats, which may alter temperature sensitivity and recruitment potential.

The second set of orthogonal contrasts compared the effects of collection year across seasonal temperatures on the likelihood of germination (Fig. 4A–C). Bog seeds collected in 2020 displayed a 2-fold germination advantage compared to 2019 following exposure to 20/10°C. However, the likelihood of germination for bog seeds collected in 2018 was 2.6 to 4.1 times greater than that of 2020 seeds across all temperatures, except at 20/10°C. Bog seeds collected in 2019 displayed a much higher capacity for germination at the highest seasonal temperatures compared to seeds collected in 2018 or 2020. In contrast to the pattern observed among bog seeds, forest seeds collected in 2020 tended to exhibit higher likelihoods of germination than seeds collected in 2018 or 2019 across seasonal temperatures. The patterns in the differences among years were also less discernible for roadside seeds than for bog seeds. For example, roadside seeds collected in 2018 and 2020 displayed a higher likelihood of germination (hazard ratio = 1.4) compared to 2019 seeds at 20/10°C. At 30/20°C, roadside seeds collected in 2019 and 2020 were more likely to germinate than 2018 seeds (hazard ratio range = 1.5 to 3.3). These differences in germination among years suggest plasticity in response to temperature which is critical for population persistence under variable climatic and habitat conditions.

In the final group of comparisons, the likelihood of germination between temperatures within the various habitats differed considerably across collection years (Fig. 5). In 2018 and 2019, seeds exposed to 25/15°C generally exhibited a higher likelihood of germination compared to seeds exposed to other temperatures, regardless of the habitat (Fig. 5A, B). In 2018, seeds exposed to lower temperatures (i.e. 20/10 and 25/15°C) always exhibited a germination advantage compared to seeds exposed to higher temperatures (i.e. 30/20 and 35/25°C). In 2019, bog seeds exposed to 30/20°C or 35/25°C displayed a higher likelihood of germination (hazard ratio range, 1.9 to 2.6) than seeds exposed to 20/10°C, as did roadside seeds exposed to 30/20°C (hazard ratio = 2.8). However, this pattern was not evident in forest seeds. In 2020, the germination advantage for seeds exposed to 25/15°C compared to seeds exposed to 20/10°C was not observed for any habitat (Fig. 5C). For seeds from all habitats, summer temperatures (35/25°C) continued to serve as a thermal limit for germination compared to lower temperatures, suggesting potential physiological constraints such as thermoinhibition (Hills and van Staden, 2003). These patterns indicate that temperature sensitivity and germination plasticity could influence seedling recruitment as temperatures increase.

### Post-germination seed viability

Total post-germination viability consisted of germinated + non-germinated but viable seeds. The original assessment approach of dissecting non-germinated seeds resulted in 18% to 96% viability for the 2018 seed lots, which varied by habitat and temperature. Using the modified approach, where non-germinated seeds were moved to optimal temperatures, resulted in more accurate post-germination viability estimates. For example, viability ranged from 55% to 96% for seeds of the 2019 collection and 87% to 98% for seeds collected in 2020 (Table 1). Roadside seeds collected in 2019 displayed viability that was about 1.0 to 1.6 times greater than seeds from bog or forest habitats at temperatures below 35/25°C (Table 1). The Kruskal–Wallis test detected a statistically significant difference in post-germination viability across years (${\chi}_2^2=12.00,P=0.0025$) but not for habitat (${\chi}_2^2=1.38,P=0.5015$) or seasonal temperatures (${\chi}_3^2=7.00,P=0.0718$). Post-hoc pairwise comparisons of viability between years using the Dwass–Steel–Critchlow–Fligner test indicated that overall post-germination viability in 2020 was significantly higher than both 2018 and 2019 (*P* < 0.05), but there was no significant difference in overall post-germination viability between 2018 and 2019.

## Discussion

Plant conservation programs increasingly rely on seed-based strategies to develop recovery actions and accomplish goals. Overcoming seed biology knowledge gaps that pose barriers to conservation is a critical step in the species recovery process. In this study, we examined the temporal influence of different parental habitats and seasonal temperatures on germination dynamics of a globally threatened forb to gain conservation-relevant perspectives on how environmental conditions can potentially influence seedling recruitment. We found considerable inter-annual variation of germination responses driven by large habitat and seasonal temperature effects.

It is well established that differences in germination responses can have genetic and environmental bases that act independently or interactively (Baskin and Baskin, 2014). The idea that the seed development environment pre-conditions differential germination responses in a trans-generational manner emerged over six decades ago (Rowe, 1964). Much of the research since then focused on how different seed collection sites for a selected species impact variable germination response (Baskin and Baskin, 2014). Our results align with this body of work, indicating clear habitat-related differences in germination dynamics. For example, roadside *H. flava* seeds displayed more robust and consistent inter-annual germination patterns over wider thermal scenarios than bog or forest seeds. Moreover, bog and forest seeds tended to display more pronounced high-temperature sensitivity than the roadside seeds.

One seed-preconditioning factor that has not received widespread attention outside of agricultural settings, but is of utmost importance for proper plant functioning, relates to parental plant mineral nutrition (Hopkins, 1995; Galloway, 2001). Baskin and Baskin (2014) summarize several studies analysing effects of various nutrients on dormancy and germination responses. Although most studies focused on nitrogen (N) effects, nutrients such as phosphorus (P), potassium (K), magnesium (Mg) and calcium (Ca) also play key roles in germination variability due to involvement in myriad seed developmental and germination processes, including ATP-binding, cell division, chlorophyll formation, enzyme catalysis, meristematic tissue growth, respiration and synthesis of dry matter reserves (Hopkins, 1995; Bewley *et al.*, 2013). Specifically, low supply or deficiencies in P, K, Mg and Ca considerably reduced germination and seedling establishment ability while balanced or increasing levels of these nutrients improved germination and subsequent seedling growth (Welch, 1999; Hejcman *et al.*, 2012; Ceylan *et al.*, 2016; Yang, 2018; Zhang *et al.*, 2020). Welch (1999) suggests that a continuous supply of Mg in sufficient amounts may be necessary for maximum seed vigour.

The enhanced germination responses of *H. flava* roadside seeds relative to bog or forest seeds coincided with higher average concentrations of P, K, Mg, Ca and larger or similar concentrations of Mn and zinc (Zn) in roadside soils (Table S1). Presently, reasons for differences in soil nutrients between the *H. flava* habitats remain unknown. However, soil pH likely plays a role, as it is known to influence the availability and uptake of soil nutrients such as N, K, Mg, Mn and Zn (Barrow and Hartemink, 2023). In strongly acidic soils (e.g. forest pH ~ 4) the uptake of these nutrients is reduced compared with moderately acidic soils (e.g. roadside pH 5.2–5.6 and bog pH ~ 5.6–5.8) (Table S1; Gardner, 2017; Barrow and Hartemink, 2023). It is also possible that these differences can be linked to fire regimes in parental habitats (Knoepp *et al.*, 2005). Research indicates that, so long as temperatures do not remain above the volatilization point for various cations, soil nutrient concentrations can increase after a fire due to deposition of ash, which facilitates re-mineralization from burned above ground vegetation (Knoepp *et al.*, 2005; Roshan and Biswas, 2023). Although fire temperatures can exceed the volatilization threshold for P and K (i.e. 774°C; Knoepp *et al.*, 2005) during burns in Florida, this is rare (Platt *et al.*, 1991; Wally *et al.*, 2006; Carrington, 2010) Temperatures that surpass volatilization thresholds for Mg, Ca, Mn or Zn (i.e. 907–1962°C) have not been reported (Platt *et al.*, 1991; Knoepp *et al.*, 2005; Wally *et al.*, 2006; Carrington, 2010; Pereira *et al.*, 2011).

Additionally, long-term mowing and the subsequent decomposition of cut biomass can contribute to improved soil nutrient concentrations, whereas soil nutrient concentrations are negatively correlated with abundance of woody vegetation and invasive species (Brunetto *et al.*, 2011; Swacha *et al.*, 2018). However, one short-term study (Christensen, 1976) did not detect a statistically significant difference in K, Mg or Ca concentration following mowing. Regardless, the periodic mowing occurring in the roadside habitat and presence of substantial woody vegetation in the forest habitat could represent possible starting points for explaining *H. flava* habitat-related nutrient differences.

Research relating soil nutrient availability or concentration to the seed biology of threatened plants is lacking. More in-depth analyses exploring relationships between germination ecology, parental plant nutrient content and partitioning, nutrient availability in soils of different habitats and management actions like mowing or prescribed fire could therefore yield important data. Such information could then refine strategies for ex situ seed propagation and seedling production, maintenance of populations at inter-situ sites, repatriating seedlings to the same habitat, and seedling translocations between habitats or to new ones. Until then, we hypothesize that *H. flava* mineral nutrition represents a foundational seed trait influencing other traits such as seed size, seed mass, germination speed, seed metabolic rate, longevity, embryo development (e.g. degree of morphological and/or morphophysiological dormancy), temperature requirement, seedling growth rate, seedling stress tolerance and dormancy breaking cues that are linked to key seed functions like persistence, germination timing and establishment (Saatkamp *et al.*, 2019).

Testing germination across simulated seasonal temperatures revealed that *H. flava* seeds are sensitive to early fall/late spring (30/20°C) and summer (35/25°C) temperatures most of the time. Portions of seed populations may be thermoinhibited yet remain viable when exposed to such high temperatures (Hills and van Staden, 2003). Conversely, simulated late fall/early spring (25/15°C) and winter (20/10°C) temperatures consistently led to comparatively improved germination responses across the seeds from different habitats and collection years. This suggests that dormancy alleviation and subsequent germination in portions of seed populations can potentially occur soon after dispersal or from a soil seed bank anytime of the year, but germination will most likely occur in the late fall through early spring. This is logical considering that the timing of natural dispersal occurs during or just prior to the onset of these cooler temperatures. Conservation practitioners may therefore want to leverage cooler temperatures when engaging in seedling production strategies.

The high degree of inter-annual and inter-habitat germination plasticity across seasonal temperatures suggests that low levels of genetic diversity, as reported for *H. flava* (Godt *et al.*, 1997; USFWS, 2016), are not necessarily detrimental when coping with current ecologically relevant temperatures. Baskin and Baskin (2023) found similar results in their synthesis (but see Aguilar *et al.*, 2019). It is plausible that such plasticity contributes to a stress-tolerant ecological strategy (Grime, 2001) for *H. flava* during the crucial seed-to-seedling transition phase. However, it is not yet clear how increases in surface temperatures brought about by predicted climate change scenarios will impact *H. flava* germination ecology. We speculate that *H. flava* germination plasticity does not appear to be neutral or maladaptive (as described in Pigliucci, 2001); at least with the habitat data and controlled settings presented in our study. However, the consequences of low genetic diversity can negatively impact the reproductive life-history stage via mechanisms like deleterious allele accumulation and lethal embryo mutations expressed during development or germination (Falk and Holsinger, 1991; Husband and Schemske, 1996; Harder *et al.*, 2012; Dussex *et al.*, 2023). Plasticity reflects a heritable trait with energetic costs for maintenance and environmental sensing (Pigliucci, 2001). Plasticity can also be limited by various factors like recent evolution of this trait (i.e. epi-phenotype problem) versus a trait integrated via natural selection (Fox *et al.*, 2019; Baskin and Baskin, 2022). Deeper analyses would need to be conducted to assess potential relationships between levels of *H. flava* germination plasticity, its genetic underpinnings and potential adaptive significance especially considering impending higher temperature stress throughout habitats of occurrence (Pigliucci, 2001; Fox *et al.*, 2019; Baskin and Baskin, 2022).

Germination plasticity between years is not unusual and has been documented extensively, given that different interannual weather patterns may influence environmental cues (e.g. temperature, soil moisture) even over small spatial scales (Baskin and Baskin, 2014). Temperatures within the three *H. flava* habitats were nearly identical within and between years. Likewise, despite some variability, precipitation was not statistically different among habitats during the flowering or seed development periods during the study period. Focusing on the comparatively reduced precipitation recorded in 2019 along with various habitat characteristics, rather than statistical significance, allows for ecologically meaningful explanations for inter-annual germination plasticity.

Forest seeds displayed the most pronounced inter-annual plasticity in terms of dormancy and germination. The large number of forest seeds that remained viable yet did not germinate across seasonal temperatures in 2019 suggests a higher proportion of the seed population expressed morphophysiological dormancy. This expression of dormancy coincided with lower overall precipitation during seed development and a sharp decrease from August through September in 2019. The decreased precipitation led to a distinct dry period for all habitats in the month prior to seed harvest. However, it is possible that *H. flava* plants encountered extensive competition for available soil moisture relative to the other habitats given the dense woody vegetation in the forest habitat. Water stress during seed development can be correlated with decreased germination capacity in subsequent generations (Baskin and Baskin, 2014; Wijewardana *et al.*, 2019; Liu *et al.*, 2020). Furthermore, physiological and morphophysiological dormancy are negatively correlated with seasonal rainfall (Baskin and Baskin, 2014; Rosbakh *et al.*, 2023). The subsequent reduction in dormancy and improved germination for forest seeds collected in 2020 aligned with higher rainfall during the seed development and maturation periods during the same year. As a result, an inverse relationship exists between the magnitude of the stimulus and phenotypic expression in successive generations (Rowe, 1964). More detailed studies on the soil, plant and seed water relations of *H. flava* could provide a more comprehensive picture of germination responses to current and future climate scenarios. Additionally, future studies examining seed morphological characteristics, such as seed mass, embryo-to-seed ratio and testa thickness, may provide additional insights to explain the observed differences in germination patterns among different habitats. These traits can influence dormancy class, water uptake and temperature sensitivity, which can determine the timing and success of germination (Baskin and Baskin, 2014; Saatkamp *et al.*, 2019).

## Conclusions

Multi-year seed biology studies provide an opportunity to capture patterns of germination plasticity inherent in seeds from wild species, and avoid the pitfalls of single-year studies, which may misrepresent estimates of focal species germination. Expanding multi-year studies to consider factors that influence pre-conditioning of germination responses may provide maximum returns. However, such studies may not always be feasible for threatened species. It may be necessary to then focus individually on the temporal influence of primary factors affecting germination responses like temperature, substrate moisture, or nutrient availability. Conservation practitioners should expect high variation in *H. flava* germination between years and different habitats. However, this degree of variation is not necessarily detrimental. Practitioners may decide to focus on cooler temperatures for plant regeneration activities and work to gain a better understanding of habitat characteristics that can influence plant fitness. More research is necessary to examine *H. flava* seed morphological characteristics from different habitats, assess germination responses to environmental conditions outside current levels, and adequately gauge the contribution of germination plasticity to potential ecotypic variation.

## Supplementary Material

Web_Material_coaf079

## Data Availability

The data used in this article will be provided by the corresponding author, A.G., upon reasonable request.
