# Correction to: Habitat, seasonal temperature and collection year drive variable germination responses in the endangered plant *Harperocallis flava*

**DOI:** 10.1093/conphys/coag032

**Published:** 2026-04-15

This is a correction to: Amber G Gardner, Héctor E Pérez, Habitat, seasonal temperature and collection year drive variable germination responses in the endangered plant *Harperocallis flava*, Conservation Physiology, Volume 13, Issue 1, 2025, coaf079, 10.1093/conphys/coaf079.

The publication date was originally errored. This is emended to read: “22 November 2025” instead of “23 April 2025” and the order of the Article history dates is corrected also respectively.

