# Supplementary material for: Habitat, seasonal temperature and collection year drive variable germination responses in the endangered plant Harperocallis flava
Source: Conserv Physiol. 2025 Nov 22;13(1):coaf079. doi: 10.1093/conphys/coaf079 (PMC12638294; doi:10.1093/conphys/coaf079)
Supplement: Web_Material_coaf079 [file web_material_coaf079.pdf]

Supplementary Tables:

Supplementary Table S1. Results from soil chemical and texture analyses. Soil samples were collected from treatment plots (open = O and sheltered =S) at each of three habitats: bog (AB), forest (FT), and roadside (RS) (modified from Gardner, 2017).

Site	pH	P (mg/Kg)	K (mg/Kg)	Mg (mg/Kg)	Ca (mg/Kg)	Mn (mg/kg)	Zn (mg/kg)	Organic Matter (%)	Sand (%)	Clay (%)	Silt (%)
ABO	5.8	0	4	0	114	0.00	0.57	1.25	94.42	3.55	2.03
ABS	5.6	1	8	1	164	0.07	0.64	1.19	80.83	6.39	12.78
FTO	4.0	2	18	0	34	0.00	2.61	5.74	81.39	4.09	14.53
FTS	3.9	1	19	8	46	0.00	1.85	4.95	83.62	4.62	11.76
RSO	5.2	3	13	35	118	0.00	1.88	2.51	87.05	4.59	8.35
RSS	5.6	5	52	81	215	0.19	2.46	2.57	86.33	5.38	8.28

Supplementary Table S2. *Harperocallis flava* fruit collection and storage information. Capsules contained 233 ± 152 (mean ± SD) seeds.

Habitat	Collection date	Capsules collected (#)	Seed lot	Storage duration (days)
Bog	8-Oct-2018	9	AB18	3
Forest	8-Oct-2018	17	FT18	3
Roadside	8-Oct-2018	14	RS18	3
Bog	23-Sept-2019	11	AB19	9
Forest	23-Sept-2019	8	FT19	9
Roadside	23-Sept-2019	19	RS19	9
Bog	7-Oct-2020	7	AB20	13
Forest	7-Oct-2020	6	FT20	13
Roadside	7-Oct-2020	16	RS20	13
